# Redo Laparoscopic Pyeloplasty in Children: Results from a Multicentric Series

**DOI:** 10.1590/S1677-5538.IBJU.2025.0077

**Published:** 2025-06-10

**Authors:** Pedro-Jose Lopez, Alejandro Calvillo-Ramirez, Ahmet Sancaktutar, Francisca Yankovic, Juan Manuel Moldes, Juan Pablo Corbetta, Luis Garcia-Aparicio, Luis H. Braga, Francisco Reed

**Affiliations:** 1 University Hospitals Rainbow Babies and Children's Cleveland OH USA Division of Urology, University Hospitals Rainbow Babies and Children's, Cleveland, OH, USA; 2 Case Western Reserve University Cleveland OH USA Case Western Reserve University, Cleveland, OH, USA; 3 Universidad de Chile Santiago de Chile Chile Universidad de Chile, Santiago de Chile, Chile; 4 Hospital Exequiel González Cortes Santiago de Chile Chile Hospital Exequiel González Cortes, Santiago de Chile, Chile; 5 Hospital Italiano de Buenos Aires Buenos Aires Argentina Hospital Italiano de Buenos Aires, Buenos Aires, Argentina; 6 Hospital de Niños JP Garrahan Buenos Aires Argentina Hospital de Niños JP Garrahan, Buenos Aires, Argentina; 7 Sant Joan de Deu Hospital Barcelona Spain Sant Joan de Deu Hospital, Barcelona, Spain; 8 McMaster Children's Hospital Hamilton ON Canada McMaster Children's Hospital, Hamilton, ON, Canada

**Keywords:** Laparoscopy, Ureteral Obstruction, Urologic Surgical Procedures

## Abstract

**Purpose:**

To describe the experience of five training centers with redo laparoscopic pyeloplasty (RLP) in children with restenosis of the uteropelvic junction (UPJ), assessing whether this approach increased or not postoperative complications or surgery failure.

**Materials and Methods:**

A retrospective, descriptive study was conducted, including 19 patients who underwent transperitoneal RLP at five independent training centers across 4 different countries between January 2009 and December 2017. All patients had previously undergone Anderson-Hynes dismembered pyeloplasty. Primary outcomes included postoperative complication rates and redo surgery failure.

**Results:**

There were 19 RLP out of 744 primary laparoscopic pyeloplasties. Median operative time was 150 minutes (interquartile range [IQR] 126.2-180), extended by 19 minutes when colon mobilization was performed. No cases required conversion to open surgery. A median postoperative analgesic requirement and length of stay of 5 and 4 days, respectively, were recorded. No major complications were reported except a single instance of temporary UPJ stenosis, which was managed with a nephrostomy tube and did not require further surgery. After a median follow-up of 17 months, we achieved a 100% success rate. A significant reduction in renal pelvis dilation was noted, with the median anteroposterior diameter (APD) decreasing from 43 mm preoperatively to 17 mm postoperatively (IQR 10–22).

**Conclusions:**

Our findings suggest that RLP remains a feasible approach in the management of restenosis of the UPJ even in such different healthcare settings, providing success rates as high as those described in primary pyeloplasty while maintaining a safety profile.

## INTRODUCTION

Ureteropelvic junction obstruction (UPJO) is the most common anatomical cause of hydronephrosis in children (1). Since its first description, laparoscopic pyeloplasty (LP) has become the standard of care for managing ureteropelvic junction obstruction (UPJO) in pediatric patients. Its widespread adoption is driven by significant benefits, including reduced postoperative pain, shorter hospital stays, and improved cosmetic outcomes. Nevertheless, the unique technical challenges in pediatric populations make LP particularly demanding for pediatric urologists (2–7).

LP is reported to have a success rate exceeding 90% (2, 4), however, adverse outcomes such as restenosis can still occur, posing additional management challenges due to potential altered anatomy and scarring at the re-stenosed UPJ. Emerging evidence suggests that redo laparoscopic pyeloplasty (RLP) remains the preferred approach in most cases of restenosis (8, 9), demonstrating a superior efficacy compared to endourological procedures such as endopyelotomy (10). Nevertheless, multicenter studies remain limited and lack detailed data, especially those involving diverse institutional settings and operative techniques.

In this study, we aimed to describe the results obtained across five teaching centers in four countries with RLP for UPJ restenosis, focusing on surgical outcomes. Additionally, we provide insights drawn from the collective expertise of the contributing authors. We hypothesized that the safety and efficacy of RLP in these teaching institutions are as high as what has been reported for primary LP, even when performed in varied healthcare settings (2).

## MATERIAL AND METHODS

### Patients and Data Collection

In our previous endeavor involving 744 LP in children across 11 participating centers over a 9-year period, 30 patients presented UPJ restenosis, requiring re-operative pyeloplasty (2); 12 were excluded from this study due to incomplete data/inadequate follow-up. Building on that work, this retrospective study focuses on children who underwent RLP. Of these, 18 cases were derived from the original cohort after meeting inclusion criteria, and one additional case involved a child who had undergone open primary pyeloplasty at one of the participating centers, yielding a total of 19 patients for inclusion. Data for this study were provided by five of the initial 11 centers. The protocol (IRB N193/2023) was approved by the ethics committees of all participating centers and data sharing was obtained.

Inclusion criteria were limited to pediatric patients who underwent transperitoneal RLP and had both baseline and postoperative ultrasonography, preoperative dynamic renal scintigraphy (DRS), and a minimum follow-up period of one year. Exclusion criteria included children with incomplete data or follow-up, or a follow-up duration of <1 year.

Recurrent UPJO was diagnosed using ultrasound criteria according to the Society for Fetal Urology (increased APD of the renal pelvis), scintigraphy findings (obstructed drainage curve on DRS), and/or symptomatology presence, primarily colicky pain. These same criteria were used to define redo surgery failure and determine the need for reoperation. Surgical success was defined as a reduction in APD on postoperative ultrasound and throughout the follow-up period, along with resolution of symptoms when present. Additionally, in seven patients where a reduction in hydronephrosis was not initially evident, success was further supported by the improvement in drainage curve on DRS.

Data collected included patients’ baseline characteristics, intraoperative parameters, and follow-up information. Baseline characteristics comprised age, sex, weight, preoperative presentation, and ultrasound parameters. For RLP-specific variables, data on primary surgery, the time interval between primary and redo surgeries, and temporizing interventions were recorded. Perioperative data included operative time (OT), operative side, surgeon, drainage, length of stay (LOS), postoperative complications, and surgery failure. Ultrasound parameters included APD, and differential renal function (DRF) assessed via DRS were also recorded when available. Postoperative complications were classified according to the Clavien-Dindo grading system, with major complications defined as grade ≥3. The primary outcomes were the rate of postoperative complications and redo surgery failure, defined as the requirement for additional redo pyeloplasty based on postoperative obstruction and persistent/worsening hydronephrosis, along with persistent symptoms. The surgical technique employed in RLP has been previously described (11).

Statistical analysis was performed with R V4.4.1 and SPSS V26 software. Descriptive statistics were used to summarize the data. The Kolmogorov-Smirnov test was employed for testing data normality. Due to nonparametric distribution, continuous variables are presented as medians and interquartile ranges (IQR), and between-group comparisons were performed with the Mann-Whitney U test. Categorical variables were expressed as frequencies with percentages, and comparisons between groups were analyzed with the Chi-square test or Fisher's exact test. Additionally, we performed a subgroup analysis incorporating a group of primary pyeloplasty from our previous series. Propensity score-matching (PSM) using the nearest neighborhood method was implemented to account for potential confounders. Therefore, the groups were balanced according to patient's characteristics, namely, age at surgery, preoperative pain and urinary tract infection (UTI), and preoperative APD and DRS. All tests were two-sided, with p <0.05 used to define significance.

## RESULTS

Over the 9-year study period, 19 RLPs were performed in 19 renal units, all through a transperitoneal approach. Of these, 18 patients initially underwent LP as primary treatment, while 1 patient was managed with open surgery. The cohort consisted of 14 males (73%) and 5 females (27%), with a median age at RLP of 56 months (32-131), and a median weight of 19.5 kg (12-31). Preoperative imaging showed progressive renal pelvis dilation, with a median APD of 43 mm (27.5-55). DRS revealed obstructed drainage curve patterns in 63% of cases, with a median renal function of 42% (30.5-45.75). The median time interval between PP and RLP was 20 months. Table-1 depicts the baseline characteristics of the studied population.

**Table 1:** Baseline and perioperative characteristics of the studied population.

Number of patients	19 (100%)
Gender	Male: 14 (73%)
	Female: 5 (27%)
Age (months)	56 (32-131)
Operative side (L/R)	14/5
Preoperative APD (mm)	43 (27.5-55)
Preoperative DRF	42 (30.5-45.7)
Follow-up (months)	19 (12-78)
Operative time (min)	150 (126.2-180)
Length of stay (days)	4 (2-5)
Length of analgesic requirement (days)	5 (2.5-6)
Duration of chemoprophylaxis (days)	4 (2-30)
External drainage (%)	11 (57.8%)
Double-J stent (%)	17 (89.4%)
Major complications (%)	1 (5.2%)
Success (%)	19 (100%)
Postoperative DRS (%)	7 (36.8%)
Postoperative DRF^a^	40 (17-48)
Postoperative APD (mm)	17 (10-22.2)

Categorical data expressed as number of events (%)

Continuous data expressed as median with interquartile range (IQR)

APD = anteroposterior diameter; DRF = differential renal function;

DRS = dynamic renal scintigraphy.

a Abstracted from seven patients.

Fourteen RLP were performed on the left side and 5 on the right. In 8 left-sided cases, the UPJ was accessed through a transmesocolic window (Supplementary Figure-1A), while in the remaining 11 (6 left-sided, 5 right-sided), the colon was mobilized. Anderson-Hynes dismembered pyeloplasty was performed in 18 cases, while one patient required a ureterocalyceal anastomosis, due to excessive scar tissue at UPJ. A percutaneous Hitch stitch was used in 9 cases to stabilize and expose the renal pelvis (Supplementary Figure- 1B). A crossing vessel was identified in 3 patients (16%).

The median OT was 150 minutes (126.2-180), with an additional 19 minutes when colon mobilization was performed. No conversions to open surgery were necessary. Median LOS was 4 days (2-5). All patients received chemoprophylaxis for a median of 4 days (2-30) and analgesics for a median of 5 days (2.5-6).

One complication (Clavien-Dindo IIIb) was observed: temporary stenosis of the UPJ in a single patient, identified by an increased APD of the renal pelvis. This case was managed with a nephrostomy tube (NT), which remained in place for 7 days. No DJ stent was placed in this patient. Kidney function was preserved, and renal pelvis dilation stabilized by the study's conclusion. The patient also developed a postoperative UTI (Clavien-Dindo II), which was successfully managed with antibiotic therapy. No additional complications were reported, and no redo surgery failures occurred in this series.

An external drain was placed in 11 patients (57.8%) to evacuate peri‐anastomotic fluid collections, kept in place for a median of 6 days (5–7). Double-J stents were inserted in 17 patients (89.4%) for a median of 8 weeks (6.5-9). Transurethral catheters were used in 18 patients (94.7%) for a median of 4 days (2-5.5).

**Figure 1:**
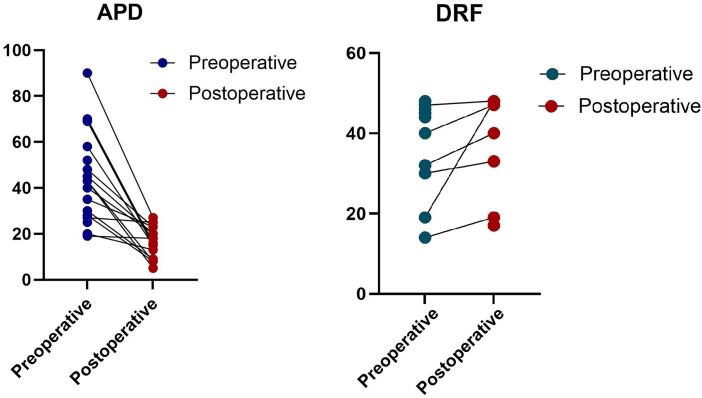
Patient-level APD and DRF changes.

With a median follow-up post-RLP of 17 months (12-86), 94.7% of patients had uneventful outcomes (1 case needed a NT). A significant reduction in renal pelvis dilation was noted, with the median APD decreasing from 43 mm preoperatively to 17 mm postoperatively (10–22). Postoperative DRS was performed in 7 patients, demonstrating improved drainage curves. Relative kidney function remained stable, with a postoperative median renal function of 40% (17-48%). Patient-level APD and DRF changes are displayed in Figure-1.

### Subgroup analysis comparing primary LP vs. RLP

Before PSM, the primary LP (PLP) group included 393 patients, while the RLP group comprised 19 patients (Table-2). Patients in the PLP group had an older median age compared to those in the RLP group (73 months vs. 56 months; p=0.005). Preoperative pain and UTI significantly differed between groups, both more common in the RLP group. RLP patients demonstrated a greater increase in preoperative APD (p=0.001) and a slightly lower DRF based on DRS results (p=0.004).

**Table 1:** Baseline and perioperative characteristics of the studied population.

	Before PSM			After PSM		
	PLP (n = 393)	RLP (n = 19)	P value	PLP (n = 19)	RLP (n = 19)	P value
Gender (M/F)	265/128	14/5	0.75	13/6	14/5	0.72
Age (months)	73 (23-130)	56 (32-131)	0.005	99 (39-144)	56 (32-131)	0.57
Weight (kg)	19.5 (10.5-36.4)	19.5 (12-31)	0.98	22 (18-45.4)	19.5 (12-31)	0.20
Preoperative pain (Y/N)	168/225	10/3	0.0001	14/5	10/3	0.83
Preoperative UTI (Y/N)	79/314	3/13	0.0001	4/15	3/13	0.86
Preoperative APD (mm)	27 (21-36)	43 (27.5-55)	0.001	34 (26.7-37.7)	43 (27.5-55)	0.10
Preoperative DRF (%)	48 (40-51.4)	42 (30.5-45.7)	0.004	48.5 (38.5-51)	42 (30.5-45.7)	0.02
Side (L/R)	255/138	14/5	0.59	11/8	14/5	0.30
Crossing vessel (Y/N)	106/287	3/15	0.33	8/11	3/15	0.09
Operative time (min)	180 (135-210)	150 (126.2-180)	0.22	165 (120-210)	150 (126.2-180)	0.53
Double-J stent (Y/N)	354/39	17/2	0.93	18/1	17/2	0.54
Duration of stent (weeks)	4 (4-6)	8 (6.5-9)	0.0001	5.5 (4-8)	8 (6.5-9)	0.03
Analgesic duration (days)	3 (2-4)	5 (2.5-6)	0.008	3 (2-4)	5 (2.5-6)	0.11
LOS (days)	2 (2-3)	4 (2-5)	0.25	3 (2-4)	4 (2-5)	0.53
Postoperative APD (mm)	13 (6-21)	17 (10-22.2)	0.20	13 (7.7-34.5)	17 (10-22.2)	0.69
Postoperative pain (Y/N)	8/308	3/12	0.0001	0/14	3/12	0.07
Postoperative UTI (Y/N)	4/386	1/16	0.07	1/16	1/16	1.00
Overall complications (Y/N)	4/161	1/15	0.49	1/11	1/15	0.78
Minor complications (Y/N)	4/161	0/16	0.52	1/11	0/16	0.21
Major complications (Y/N)	0/165	1/15	0.001	0/11	1/15	0.39

APD: anteroposterior diameter; DRS: dynamic renal scintigraphy; LOS: length of stay; PLP: primary laparoscopic pyeloplasty; PSM: propensity score-matching; RLP: redo laparoscopic pyeloplasty; UTI: urinary tract infection.

Among patients who received a DJ stent, the median time to stent removal was significantly longer in the RLP group (8 weeks vs. 4 weeks; p=0.0001). Major complications were also more frequently observed in the RLP group (p=0.001).

Following PSM, both groups were balanced with 19 individuals each. Preoperative DRF remained slightly lower in the RLP group (p=0.02), as did the longer median time to stent removal (0.03). However, no significant differences were observed in age, pre- and postoperative APD, or major complications between the two groups.

## DISCUSSION

In the present study, 19 patients with UPJ restenosis were managed with RLP. After a median follow-up of 17 months, only one postoperative complication was observed, and no cases of redo surgery failure were reported, yielding a 100% success rate. Previous studies on RLP in children similarly reported high success rates and low complication rates. For instance, a 22-patient series documented a 91% success rate with minimal complications (12), while Li et al. reported no surgical failures in their 10-case series, despite three postoperative complications (13). These findings align with the current study, which demonstrated no instances of redo surgery failure and fewer postoperative complications. Furthermore, we observed similar results to those reported for conventional pyeloplasty, whether performed using minimally invasive surgery (MIS) or open techniques (2–8, 14, 15).

MIS has gained significant traction in pediatric urology, facilitated by advancements in instrumentation and growing expertise across training centers. For instance, the da Vinci Single Port platform has been increasingly adopted recently, with emerging evidence supporting its use in the management of UPJO (16). Despite these advances, up to 10% of patients undergoing primary pyeloplasty experience anastomotic restenosis, representing an ongoing clinical challenge (17). Both laparoscopic and robotic approaches have shown promising outcomes in the redo setting, with reported success rates exceeding 80% (12,18–20). In contrast, primary balloon endopyelotomy has shown approximately a 65% success rate, which is lower than that of RLP, particularly in patients with complete failure of the initial anastomosis (21).

As laparoscopic technology becomes more affordable and accessible, the adoption of this technique has become feasible even in resource-limited settings, unlike robotic surgery, which remains cost-prohibitive in many regions (2). Laparoscopy thus represents a viable option for managing failed primary pyeloplasties, offering the advantages of MIS at a lower overall cost.

Achieving success in both primary and redo pyeloplasty requires attention to several key technical considerations. We highlight the importance of adequate tissue irrigation and meticulous dissection down to healthy tissue. In cases where this is not feasible, ureterocalyceal anastomosis serves as a viable alternative (19), as in one case of this series. It should be noted that no matter how thin the renal parenchyma is, bleeding from this technique can complicate the anastomosis, necessitating aspiration via a fourth trocar/trocar-less approach. Watertight suture and tension-free anastomosis are other crucial aspects for a successful RLP, even though these may be more challenging to achieve in a redo setting (13, 22). One advantage of performing this suture laparoscopically is the enhanced visualization provided, ensuring tight closure and improved anchoring.

Colon mobilization plays a crucial role in identifying possible aberrant vessels that may have been missed during previous surgery, as observed in 16% of our population. In redo pyeloplasty, significant fibrosis is often encountered at the UPJ. We stress that colon mobilization improves the surgical field and facilitates the procedure by allowing dissection to begin from healthy ureteral tissue, facilitating the procedure, even though almost half of the left-sided RLP in this series (n=8) could be performed transmesocolically.

One recognized limitation of laparoscopy is the longer OT. However, it has been shown that OT decreases with experience as surgeons move beyond the learning curve (19,22,23). All centers included in this study are teaching institutions with considerable expertise in MIS, where LP is a standard practice. This might explain our median OT of 150 minutes. Interestingly, the OT observed in this study is shorter than that reported in our initial multicenter series, contrary to initial expectations (2). This could be due to less centers, concentrated cases and better learning curve.

On the other hand, a recent meta-analysis evaluating RLP in children suggested that shorter LOS was influenced by studies conducted in countries where surgeons may be inclined to shorter stays due to cost constraints (20). The 84% of the studied population in the current study was provided by South American institutions, where longer hospital stays may not present the same financial burden as in US or European countries. This cultural and healthcare context may partially explain the longer LOS, rather than patient condition.

The absence of significant bleeding and postoperative UTI in uneventful patients are other aspects that increase the likelihood of a successful RLP. Both factors are known to exacerbate tissue inflammation and increase the risk of secondary fibrosis (15, 21). Indeed, the only patient requiring subsequent management with NT experienced a UTI during the early postoperative period.

The use of DJ stents in urological procedures remains a subject of debate with pyeloplasty not being the exception, though their benefits in providing anastomotic support and preventing edema have been described in the latter (24, 25). In our current work, a DJ stent was placed in 89.4% of cases, a rate similar to the 87% observed in our initial series (2). Despite the widespread use of DJ stents, we observed low rates of UTI and major complications in both our initial and current series.

Our study reinforces previously reported findings on RLP, demonstrating its feasibility and safety in managing UPJO recurrence, with high success rates. However, several limitations must be acknowledged. First, the retrospective and observational nature of the study introduces potential observer bias and confounding. Additionally, a substantial number of patients were excluded due to incomplete data and/or a follow-up duration of <1 year at the time of study inception, which reduced the final sample size, representing a major drawback to our study—though it is important to note that these excluded patients had successful surgical outcomes. This may be further accentuated by the uncommon nature of UPJO recurrence requiring RLP. Although a subgroup analysis comparing PLP and RLP was performed, not all variables were uniformly recorded across patients, limiting statistical power and potentially affecting the robustness of comparisons. Furthermore, the short follow-up period restricted our ability to assess long-term outcomes, thereby limiting the generalizability of our findings to prolonged recovery timelines. Despite these limitations, our study provides valuable insights and further supports the feasibility and effectiveness of RLP, particularly when performed in experienced centers with established expertise in PLP.

## CONCLUSIONS

The findings in the current study demonstrate that RLP remains a feasible approach in the management of restenosis of the UPJ, with success rates similar to those reported in PLP, without increasing the risk of complications. Surgeons with enough expertise performing PLP may not be discouraged to pursue this approach whenever UPJ restenosis requires redo surgery. Future research should implement longer follow-ups across larger sample sizes to draw more robust conclusions, especially to consult parents.

## Figures and Tables

**Supplementary Figure 1A:**
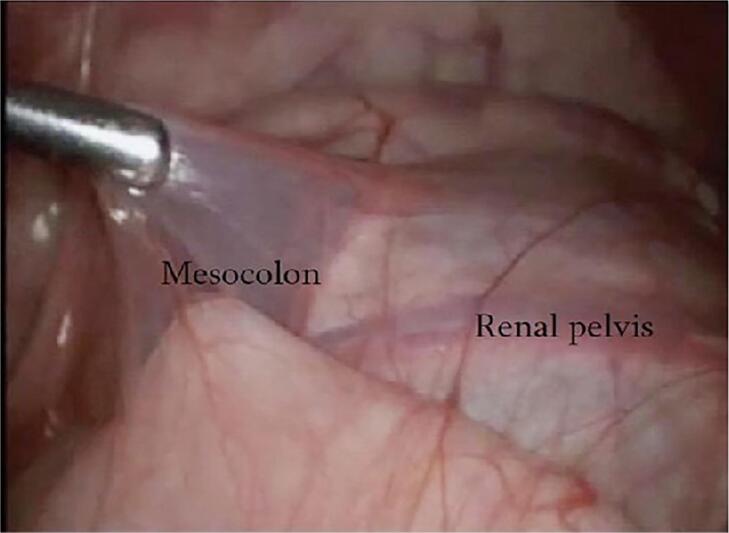
Transmesocolic access.

**Supplementary Figure 1B:**
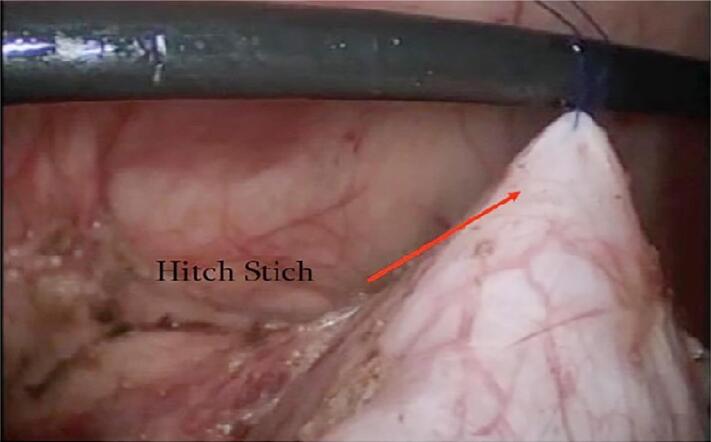
Percutaneous hitch stitch.

## ABBREVIATIONS

APD: Anteroposterior diameter
DRF: Differential renal function
DRS: Dynamic renal scintigraphy
LOS: Length of stay
LP: Laparoscopic pyeloplasty
MIS: Minimally invasive surgery
NT: Nephrostomy tube
OT: Operative time
PLP: Primary laparoscopic pyeloplasty
PSM: Propensity score-matching
RLP: Redo laparoscopic pyeloplasty
UPJ: Ureteropelvic junction
UPJO: Ureteropelvic junction obstruction
UTI: Urinary tract infection

## References

[1] Dias CS, Silva JMP, Pereira AK, Marino VS, Silva LA, Coelho AM (2013). Diagnostic accuracy of renal pelvic dilatation for detecting surgically managed ureteropelvic junction obstruction. J Urol.

[2] Echeverria P, Reed LF, Gatti JM, Braga LH, Cherian A, Garcia-Aparicio L (2023). Latitudes and attitudes: A multinational study of laparoscopic pyeloplasty in children. J Pediatr Urol.

[3] Krajewski W, Wojciechowska J, Dembowski J, Zdrojowy R, Szydełko T (2017). Hydronephrosis in the course of ureteropelvic junction obstruction: An underestimated problem?. Adv Clin Exp Med.

[4] Autorino R, Eden C, El-Ghoneimi A, Guazzoni G, Buffi N, Peters CA (2014). Robot-assisted and laparoscopic repair of ureteropelvic junction obstruction: a systematic review and meta-analysis. Eur Urol.

[5] van der Toorn F, van den Hoek J, Wolffenbuttel KP, Scheepe JR (2013). Laparoscopic transperitoneal pyeloplasty in children from age of 3 years: our clinical outcomes compared with open surgery. J Pediatr Urol.

[6] Piaggio LA, Franc-Guimond J, Noh PH, Wehry M, Figueroa TE, Barthold J (2007). Transperitoneal laparoscopic pyeloplasty for primary repair of ureteropelvic junction obstruction in infants and children: comparison with open surgery. J Urol.

[7] Ravish IR, Nerli RB, Reddy MN, Amarkhed SS (2007). Laparoscopic pyeloplasty compared with open pyeloplasty in children. J Endourol.

[8] Alhazmi HH (2018). Redo laparoscopic pyeloplasty among children: A systematic review and meta-analysis. Urol Ann.

[9] Chiancone F, Fedelini M, Pucci L, Meccariello C, Fedelini P (2017). Laparoscopic management of recurrent ureteropelvic junction obstruction following pyeloplasty: a single surgical team experience with 38 cases. Int Braz J Urol.

[10] Ceyhan E, Dogan HS, Tekgul S (2020). Our experience on management of failed pediatric pyeloplasty. Pediatr Surg Int.

[11] Reed F, López PJ (2014). Laparoscopic Pyeloplasty.

[12] Al-Hazmi H, Peycelon M, Carricaburu E, Manzoni G, Neel KF, Ali L (2020). Redo Laparoscopic Pyeloplasty in Infants and Children: Feasible and Effective. Front Pediatr.

[13] Li J, Yang Y, Li Z, Fan S, Wang X, Yang Z (2022). Redo laparoscopic pyeloplasty for recurrent ureteropelvic junction obstruction: Propensity score matched analyses of a high-volume center. Front Pediatr.

[14] Rai A, Hsieh A, Smith A (2022). Contemporary diagnosis and management of pelvi-ureteric junction obstruction. BJU Int.

[15] Andolfi C, Lombardo AM, Aizen J, Recabal X, Walker JP, Barashi NS (2022). Laparoscopic and robotic pyeloplasty as minimally invasive alternatives to the open approach for the treatment of uretero-pelvic junction obstruction in infants: a multi-institutional comparison of outcomes and learning curves. World J Urol.

[16] Ditonno F, Franco A, Manfredi C, Chow AK, Vourganti S, Cherullo EE (2023). Single Port Robotic Pyeloplasty: early single-center experience. Int Braz J Urol.

[17] Dy GW, Hsi RS, Holt SK, Lendvay TS, Gore JL, Harper JD (2016). National Trends in Secondary Procedures Following Pediatric Pyeloplasty. J Urol.

[18] Moscardi PRM, Barbosa JABA, Andrade HS, Mello MF, Cezarino BN, Oliveira LM (2017). Reoperative Laparoscopic Ureteropelvic Junction Obstruction Repair in Children: Safety and Efficacy of the Technique. J Urol.

[19] Powell C, Gatti JM, Juang D, Murphy JP (2015). Laparoscopic Pyeloplasty for Ureteropelvic Junction Obstruction Following Open Pyeloplasty in Children. J Laparoendosc Adv Surg Tech A.

[20] Chandrasekharam VVS, Babu R (2022). A systematic review and metaanalysis of open, conventional laparoscopic and robot-assisted laparoscopic techniques for re-do pyeloplasty for recurrent uretero pelvic junction obstruction in children. J Pediatr Urol.

[21] Abdrabuh AM, Salih EM, Aboelnasr M, Galal H, El-Emam A, El-Zayat T (2018). Endopyelotomy versus redo pyeloplasty for management of failed pyeloplasty in children: A single center experience. J Pediatr Surg.

[22] Soledad Celis Pedro-José López (2012). Transperitoneal Laparoscopic Pyeloplasty: more Room, More Feasibility. Universidad de Chile, Santiago, Chile. Dialogues in Pediatric Urology.

[23] Soledad Celis Reed F (2012). Best Way of Training: How To Improve Learning Curve. Universidad de Chile, Santiago, Chile. Dialogues in Pediatric Urology.

[24] Calvillo-Ramirez A, Angulo-Lozano JC, Del Rio-Martinez CJ, Esparza-Miranda LA, Perez Rodriguez Garcia G, Macías-Cruz HM (2024). Safety and effectiveness of preoperative stenting compared to non-stenting in ureteroscopy for urinary stone disease: a meta-analysis of comparative studies. World J Urol.

[25] Elmalik K, Chowdhury MM, Capps SNJ (2008). Ureteric stents in pyeloplasty: a help or a hindrance?. J Pediatr Urol.

